# Editorial: Dissecting the immunological, pathological, and clinical aspects of autoimmune gastritis and its neoplastic complications

**DOI:** 10.3389/fimmu.2022.1070250

**Published:** 2022-10-27

**Authors:** Marco Vincenzo Lenti, Bruno Annibale, Antonio Di Sabatino, Edith Lahner

**Affiliations:** ^1^ Department of Internal Medicine and Medical Therapeutics, University of Pavia, Pavia, Italy; ^2^ First Department of Internal Medicine, Fondazione Istituto di Ricovero e Cura a Carattere Scientifico (IRCCS) Policlinico San Matteo, Pavia, Italy; ^3^ Department of Medical-Surgical Sciences and Translational Medicine, Sant’Andrea Hospital, University Sapienza, Rome, Italy

**Keywords:** autoimmunity, dysplasia, gastritis, neuroendocrine, tumor, vitamin B12

Autoimmune gastritis (AIG) is a non-self-limiting, chronic, inflammatory disorder of the stomach causing progressive atrophy of the oxyntic mucosa which, in turn, leads to vitamin B12, iron, and other micronutrient deficiencies and eventually to neoplastic progression, namely gastric cancer and type 1 gastric neuroendocrine tumors (1). Our understanding about AIG epidemiology, pathophysiology, and clinical aspects has been deeply advanced over the last decades, although many gaps remain to be filled. Notably, this Research Topic includes five original and innovative articles, three of them focusing on inflammation-related issues and due of them focusing on items related to neoplastic risk.

The first study (Lenti et al.) provides an answer to an important, unanswered question of the natural history of AIG, namely whether there is a histopathological marker of potential AIG, reliably predicting the evolution of anti-parietal cells antibody (APCA)-positive patients and without gastric histopathological alterations into overt AIG, i.e. atrophy of the oxyntic mucosa. A previous study showed that the definitive evolution from potential into overt AIG may occur in about 50% of patients over a median observation period of eight years (2). By thoroughly assessing the intraepithelial lymphocyte (IEL) infiltration in the gastric corpus of potential AIG patients as compared to patients controls (H. pylori gastritis, celiac disease, healthy controls), they showed that and increased deep CD3+ IEL infiltration of the corpus oxyntic mucosa was characteristic of AIG patients before and after developing overt AIG, thus representing a histopathological marker of potential AIG. This finding needs confirmation by larger studies, because the detection of AIG in a very early phase may hopefully offer therapeutic possibilities and early vitamin B12 supplementation, thus preventing complications.

Other two studies provided novel data on the inflammatory pathogenetic mechanisms in AIG. The proton pump of parietal cell H+/K+-adenosine triphosphatase (ATPase) is the key autoantigen recognized in both AIG and experimental autoimmune gastritis (EAIG) (Della Bella et al.). Autoreactive T helper 1 (Th1 cells) secreting IFN-gamma play a pivotal role in AIG (Della Bella et al.), but the role of T17-driven inflammation in AIG has not been addressed so far. This study showed that IL-17A and IL-17F are produced *in vivo* by the lamina propria mononuclear cells (LPMCs) of the gastric mucosa of AIG patients, following activation with the H+/K+-ATPase, and serum IL-17A, IL17F, IL-21, and IL-17E levels are significantly higher in AIG patients. These results add a new piece of the puzzle to the complex AIG pathogenesis, showing a T17 signature in AIG. Notably, the long-term T17 responses have been associated gastric carcinogenesis (3), hence T17 cytokines might be taken into consideration as potential markers of cancer risk in AIG.

A further paper focuses on pernicious anemia (PA), known as the end-stage clinical manifestation of AIG. As a consequence of long-standing vitamin B12 malabsorption due to intrinsic factor deficiency, this megaloblastic anemia may occur, and - when untreated – may lead to serious, and sometimes irreversible, neurological alterations (1). As for AIG, the immunopathogenic mechanisms of PA are characterized by autoreactive T cells specific for the H+/K+-ATPase of parietal cells, but in PA also for the intrinsic factor (Della Bella et al.). The role of the IL-20 cytokine subfamily, constituting a key link between the immune system and epithelial tissues and exerting essential functions in innate mucosa immunity, has not been addresses so far in PA. The results of this study showed that that IL-19 is an important cytokine in the immunopathogenesis of PA, as IL-19 levels are higher in the serum of PA patients when compared to patients with iron deficiency anemia without AIG. Additionally, LPMC obtained from PA patients produced higher levels of IL-19 when compared to controls, together with IL-17 and TNF-alpha, as previously reported (Della Bella et al.), able to induce IL-19 expression. This innovative finding provides further knowledge on the inflammatory mechanisms in AIG with PA, starting to define a peculiar immunopathogenic which in future may be helpful to explain why a subset of patients develop this long-term hematological complication.

Another article addressed the important issue of the role of miRNAs in AIG, as compared to other types of atrophic gastritis. miRNAs, a group of small, single-stranded non-coding RNAs, are proposed as biomarkers of neoplastic progression and possible therapeutic targets in immune-mediated diseases (Zingone et al.). In AIG still there is a lack of reliable molecular biomarkers of gastric neoplastic progression. The interesting findings of this study showed that amongst the miRNAs known to be involved in gastric cancer, miR-21 was over-expressed irrespective of the etiology of gastric atrophy, and miR-142 and miR-223 were under-expressed in atrophic gastritis, when compared to controls. This finding raises two considerations: first, once gastric atrophy is established, the mechanisms of neoplastic progression seem occur independently from the initial trigger of the atrophic damage, and second, miRNAs merit further attention as they seem promising serological markers to assess the individual risk of gastric neoplastic progression. This would allow tailoring a proper endoscopic surveillance.

Finally, a case-control study addressed the potential risk factors for the development of gastric cancer in AIG, that is the use of proton pump inhibitors (PPIs) prior to the diagnosis of AIG. Two recent papers showed an increased risk of gastric cancer in patients on PPIs (Dilaghi et al.). In patients with AIG, due to the presence of oxyntic atrophy, gastric acid secretion either reduced or completely lacking. For this reason, when AIG is diagnosed, PPIs are withdrawn, as their use is irrational. By comparing a group of AIG patients who at long-term follow-up developed gastric neoplastic lesions (dysplasia or cancer) and a gender-, age-, and follow-up-matched group of AIG patients who did not develop gastric neoplasia, a positive association of PPIs use before AIG diagnosis was shown in AIG patients with neoplastic lesions (OR 9,6), while other variables/confounders, as antiplatelets/anti-aggregants, age, family history of gastric cancer, or smoking habit, had no role. The reason for this association is not clear, but it could be due to the deep pharmacological acid suppression in an already achlorhydric stomach, worsening the inflammation-driven damage and easing the progress of the atrophic and neoplastic damage. It has been shown that AIG is often diagnosed with delay with respect to the first clinical presentation (4) and dyspeptic symptoms are often empirically treated with PPIs, an approach which may be harmful when there is an underlying, undiagnosed AIG. Thus, this study raises two important points, namely the indication of PPIs treatment should periodically be checked and these drugs should be withdrawn when not necessary to avoid harmful consequences; in patients with AIG, PPIs should always be withdrawn; and AIG patients who used PPIs prior to diagnosis should be carefully monitored for the higher risk of gastric neoplasms.

In conclusion, this Research Topic provides new information filling some important gaps in the knowledge on AIG, regarding the immunopathogenesis and the gastric neoplastic risk. But, still far from being able to answer all the open questions on this intriguing and complex condition, further research is needed to get a complete picture of AIG to optimize management of patients.

## Author contributions

All authors participated in the drafting of the paper, made critical revision of the manuscript for important intellectual content, and provided approval of the final submitted version.

## Conflict of interest

The authors declare that the research was conducted in the absence of any commercial or financial relationships that could be construed as a potential conflict of interest.

## Publisher’s note

All claims expressed in this article are solely those of the authors and do not necessarily represent those of their affiliated organizations, or those of the publisher, the editors and the reviewers. Any product that may be evaluated in this article, or claim that may be made by its manufacturer, is not guaranteed or endorsed by the publisher.
